# Pessaries

**Published:** 1884-06

**Authors:** 


### Pessaries.

Undoubtedly a woman wearing a pessary should not be sent away ignorant of its presence, and without any directions. She should, therefore, be informed that such an instrument has been inserted, and she should be given certain directions. Thus, it is advisable at once to tell her that it is well to wash out the vagina once or twice a day with simple water, which will prevent secretions from accumulating, decomposing, and causing an unpleasant smell (which in some cases is bad enough to suggest the presence of cancer); she should also be told that soreness, itching, or profuse discharge, indicates that the pessary should be seen to, and, generally, that it should not be worn without being seen to three or four times a year.

It is also usually advisable that the doctor should satisfy himself in a week or so that the pessary is doing good, and doing no harm, and then, having once started the treatment, the patient should be left to test its efficacy. Now this test implies the removal of some symptoms or symptom, which may justly be attributed to some former morbid condition, and it also implies the locomotion of the patient. Generally speaking, a patient lying down is better without a pessary, whatever displacement is present; thus it is rare for even complete procidentia not to reduce itself, or become much smaller, when the patient lies down, and the symptoms of partial descent, which (if there are any symptoms at all) will include almost certainly a sense of weight and dragging pain in one or other iliac fossa, will disappear or become greatly diminished in recumbent position. A pain which is better when the patient is standing and worse when she is lying should be regarded with suspicion if supposed to be due to descent or displacement; it is probably nothing of the kind. Thus it is to relieve pains by standing that pessaries are most generally useful. If this is not effected, the uterus may be unquestionably in the "normal" position, but the pessary is useless, and if useless, injurious. Thus the proper use of pessaries is—first, in most cases after the insertion of the pessary to get the patient on her legs; secondly, to satisfy oneself in a few days that it is doing good and is doing no harm; but as soon as both these objects are attained to, send the patient away with the above directions. It should not be the task of months to fit a woman with a pessary any more than with a truss. The following are not instances of the proper use of pessaries: To keep the patient in bed for long periods, wearing a pessary; to see her every day, every other day, twice a week for weeks, months, or years. Perhaps such visits are not made to the patient but to the pessary. However that may be, it is not the pessary but the patient who has to pay. What should we say of a surgeon who called for months to see a patient to whom he had given a wooden leg or a truss, and who kept him in bed for long periods, or an oculist who had fitted a patient with spectacles and saw him every day for months whether the spectacles seemed to suit him or not? It is true that the pessary is a truss in the dark, but that is no reason why the management of a pessary should be a deed of darkness. Recent investigations have shown that the whole question of displacements has to be reconsidered. It cannot be too widely or too dogmatically stated that prolonged treatment by pessaries, such as we have described, is quite inadmissible and unnecessary, injurious not only to the patient—i. e., to her *morale* as well as her purse—but also in the best sense to the practitioner, and if to the patient and practitioner, then to the public and the profession. It should also be realized that a pessary is a mere form of truss, and that its operations, though removed from the general view, are not occult Ill-treatment bids fair to bring this useful form of truss into disrepute, and we are daily expecting to meet the practitioner whose sensitiveness is such that he shrinks from a cure whose name he has learnt to mistrust and dislike, but we feel bound to say we have not come across him yet.—*London Lancet.*

